# Comprehensive geriatric assessment for older women with early breast cancer – a systematic review of literature

**DOI:** 10.1186/1477-7819-10-88

**Published:** 2012-05-17

**Authors:** Ruth M Parks, Radhika Lakshmanan, Linda Winterbottom, David AL Morgan, Karen Cox, Kwok-Leung Cheung

**Affiliations:** 1 Division of Breast Surgery, University of Nottingham, Nottingham, UK; 2 Nottingham Breast Institute, Nottingham University Hospitals, Nottingham, UK; 3 Department of Oncology, Nottingham University Hospitals, Nottingham, UK; 4 School of Nursing, University of Nottingham, Nottingham, UK; 5 Division of Breast Surgery, School of Graduate Entry Medicine & Health University of Nottingham, Royal Derby Hospital, Uttoxeter Road, Derby, DE22 3DT, UK

**Keywords:** Comprehensive geriatric assessment, Primary, Operable, Breast cancer, Early

## Abstract

**Background:**

The Comprehensive Geriatric Assessment (CGA) is an analytical tool increasingly implemented in clinical practice. Breast cancer is primarily a disease of older people; however, most evidence-based research is aimed at younger patients.

**Methods:**

A systematic review of literature was carried out to assess the use of CGA in older breast cancer patients for clinical decision making. The PubMed, Embase and Cochrane databases were searched.

**Results:**

A total of nine useful full text article results were found. Only five of these were exclusively concerned with early breast cancer; thus, studies involving a variety of cancer types, stages and treatments were accepted, as long as they included early breast cancer.The results comprised a series of low sources of evidence. However, all results shared a common theme: the CGA has a use in determining patient suitability for different types of cancer treatment and subsequently maximizing the patient’s quality of life.

**Conclusions:**

There is not yet sufficient high level evidence to instate CGA guidelines as a mandatory practice in the management of breast cancer, due to the heterogeneity of available studies. More studies need to be conducted to cement current work on the benefits of the CGA. An area of particular interest is with regard to treatment options, especially surgery and chemotherapy, and identifying patients who may be suitable for these treatments.

## Background

The Comprehensive Geriatric Assessment (CGA) is a multidisciplinary management tool aimed at determining an older person’s medical, psychological and functional capability [1].

Current evidence regarding breast cancer is mainly appropriate to younger patients (≤65 years) as older patients are often excluded from clinical trials [2,3]. There is a need for further research focusing solely on older patients, or by stratification of patients by age, to allow for accurate treatment guidelines.

Disadvantages of CGA include additional time of implementation and limited consensus regarding methodology, evaluation and utilization [3]. Comprehensive geriatric assessment generally consists of a few major components, including: medical assessment of current diagnoses, medications and nutritional status; assessment of physical function; psychological evaluation to determine patient mentality and mood; and social and environmental assessments [1].

Currently, CGA is not used routinely in breast cancer patients worldwide; however, three main areas where CGA could potentially be implemented include the following.

### Assessing fitness for treatment

Since age alone may not be an accurate predictor of treatment outcome [4], CGA could assist in distinguishing between those who should be given more invasive treatments after taking into consideration tumor type and different treatment options.

### Assessing appropriateness of treatment

Greater comorbidity increases risk of death from causes other than breast cancer [5,6]. Consequently, older patients may feel the benefit of surgery for breast cancer is not worthwhile, though with modern surgical and improved anesthetic techniques, fewer patients are now deemed unfit for surgery. On the other hand, there may be situations where non-operative therapies (for example, primary endocrine therapy) or even no treatment may be considered preferable due to a number of factors, some of which are related to frailty and/or co-morbidities.

### Identifying deficits in health

There is the need for identification of patients with confounding health problems, social needs or other issues that may have otherwise remained undetected [7], which could impact on the management of the patient’s cancer.

Assessment in these areas allows establishment of targeted treatment plan specific to the individual patient, leading to potential benefits, such as optimization of medical treatment; improved diagnostic accuracy and prognosis; maintained function; and improved quality of life (QOL) [8-11].

The aim of this systematic literature review was to analyze current evidence regarding CGA in early breast cancer and highlight possible areas for further research.

## Methods

**Figure 1:**
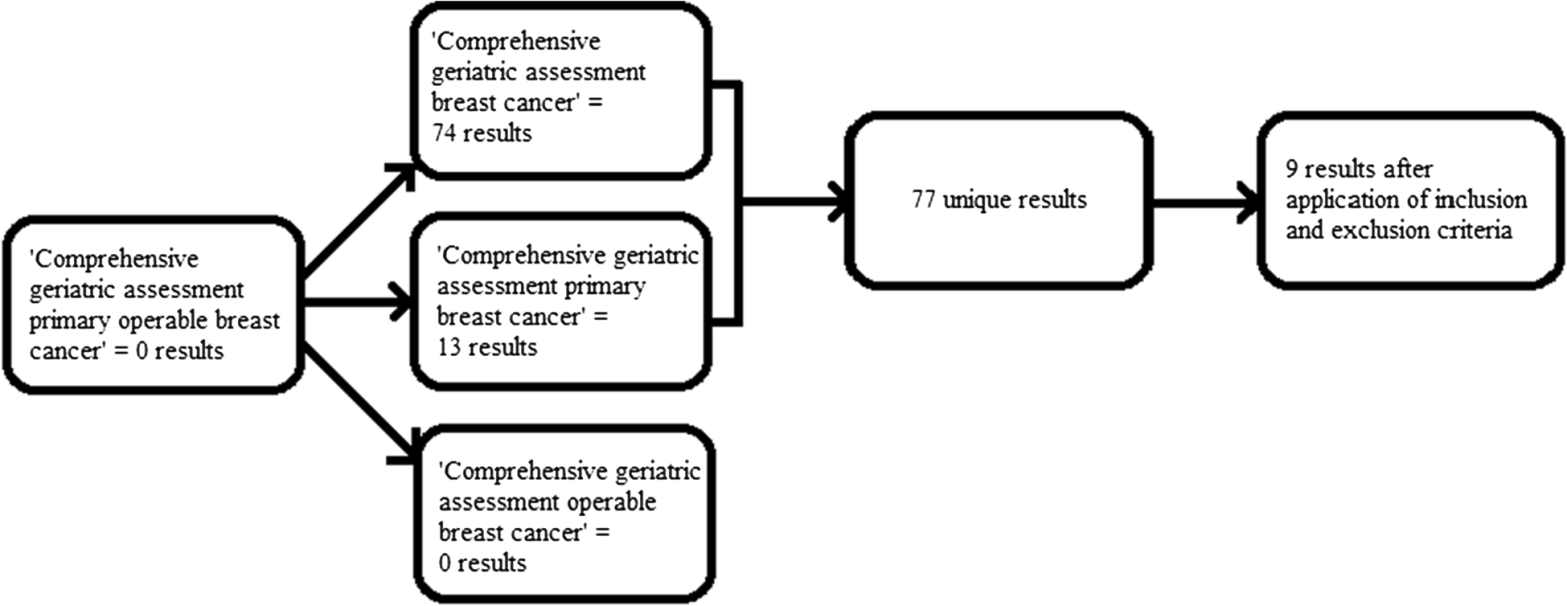
Selection of articles for review.

Three online databases were searched for relevant literature, including full-text articles and abstracts. These were PubMed, Embase and the Cochrane Library, which cover most clinical studies with high level evidence. The following key words were used: comprehensive geriatric assessment, breast cancer, primary, operable. Studies published in English in the past 10 years (January 2001 to September 2011, as far as access was allowed) were included. Studies were excluded if: a form of geriatric assessment was not used in the methodology; there was no relation to cancer; or no early breast cancer patients were included (Figure 1).

The search limits yielded nine full-text articles (Table 1).

**Table 1:** Results from full-text articles

**Date**	**Study**	**Context**	**L**	**N**	**Age**	**Type**	**Stage**	**Uniqueness of study**	**Findings**
Jan 2004	Extermann M *et al.*[12]	Single center pilot study assessing role of CGA in oncological treatment.	3	15	≥70	Breast	55% Stage I, 45% Stage II	QOL was measured by the Functional Assessment of Cancer Treatment-Breast tool. Functional status was assessed by ADL, IADL, ECOG-PS, GDS, MNA, Charlson Comorbidity Index, CIRS-G.	CGA with follow-up can extend quality of life in these patients from treatment and prognostic aspects.
Nov 2005	Hurria A *et al.*[13]	Feasibility study concerning a cancer-specific CGA.	3	43	≥65	25% breast, 18% CRC, 38% lung, 20% lymphoma	5% Stage 1, 10% Stage II, 18% Stage III, 68% Stage IV	Specifies use of ADL, IADL, KPS, Timed Up and Go, BOMC, HADS, MOS, Seeman and Berkman Social Ties, BMI, % unintentional weight loss in the last six months in a CGA that is specific for cancer. Specifies ethnicity of participants as 90% white, 10% black.	The cancer-specific CGA can be completed by the majority of patients to provide reliable and valid results.
Dec 2006	Pope, D *et al.*[14]	Study of Geriatric Assessment in older patients with operable cancer.	3	460	>70	216 breast, 146 GIT, 71 GUT, 27 other	For breast cancer: 48% Stage 1, 39.3% Stage 2, 12.7% Stage 3.	Specified use of PACE, an advanced form of CGA using BFI, ECOG-PS, IADL, GDS, MMSE, ASA and SIC.	Geriatric Assessments can be a useful tool in evaluating fitness for surgery in the older cancer patient.
Jan 2008	Albrand, G and Terret, C [15]	Single-center review of assessment of breast cancer in the older.	3	76	>70	Breast	Primary	Specific for breast cancer. Specifies use of CIRS-G score as part of CGA.	CGA should be performed before any treatment decisions are made. More trials need to be conducted involving older patients, to determine efficacy of chemotherapy in older breast cancer patients.
March 2010	Gironés, R *et al.*[16]	Single-center experience of use of CGA for breast cancer patients.	3	91	>70	Breast	26% Stage I, 58% Stage II, 16% Stage III	Specific for breast cancer, specifying use of ADL, IADL, ECOG PS, GDS, Charlson comorbidity index, BMI, Balducci criteria for frailty in CGA.	Function and independence in older breast cancer patients with co-morbidities can be preserved by use of CGA. CGA is often too time consuming to be practically assessed in an oncology setting.
Jul 2010	Molina- Garrido, M and Guillén-Ponce, C [17]	Single center comparison of two frailty screening tools and CGA.	3	41	≥65	Breast	53.7% Stage I, 41.5% Stage II, 2.4% Stage III, 2.4% Stage IV	Specific for breast cancer. Specifies use of ADL, IADL, Charlson comorbidity index, Pfeiffer test for cognition and NSI.	Patients who had a score indicative of frailty had scores in CGA suggesting poorer physical function, malnutrition and cognition.
Jul 2011	Barthélémy, P *et al.*[18]	Single center retrospective review of recommendation of adjuvant chemotherapy to older breast cancer patients	3	192	>70	Breast	Primary	Specific for primary breast cancer only. Specifies use of ADL, IADL, ECOG PS, GDS, BMI, MNA, CIRS-G.	Some components of CGA may be able to determine its use in predictability of chemotherapy use.
Jul 2011	Lazarovici, C *et al.*[19]	Single center investigation into what components of CGA correlate to need for geriatric referral within older cancer patients	3	65	≥71	Breast (29%), lung (24.6%), colorectal (7.6%)	60% had metastatic disease	Specifies use of ADL, IADL, MMSE, BMI, CIRS-G	Patients who had CGA before they had made their cancer treatment decision were more likely to receive an altered treatment plan.
Aug 2011	Hurria, A *et al.*[20]	A multicenter trial to assess use of CGA in determining chemotherapy toxicity in older cancer patients	3	700	≥65	Lung (29%), GI (27%), gynaecological (17%), breast (11%), genitourinary (10%), or other (6%)	5% Stage I, 12% Stage II, 22% Stage III, 61% Stage IV	Same CGA format used in previous study by Hurria *et al.*[13]	Some components of the CGA can be used to assess potential toxicity of chemotherapy in this group of older cancer patients.

ADL, Activities of Daily Living; ASA, American Society of Anesthesiologists grade; BFI, Brief Fatigue Inventory; BMI, Body Mass Index; BOMC, Blessed-Orientation Memory Concentration test; CGA, Comprehensive Geriatric Assessment; CIRS-G, Cumulative Illness Rating Scale – Geriatrics; CRC, Colorectal cancer; ECOG-PS, Eastern Cooperative Oncology Group Performance Status Scale; GDS, Geriatric Depression Scale; GIT, Gastro-intestinal Tract; GUM, Genito-urinary Tract; HADS, Hospital Anxiety and Depression Scale; HHC, Home Health Care; IADL, Instrumental Activities of Daily Living; KPS, Karnofsky Performance Status; L, Level of evidence; MMSE, Mini-mental state examination; MNA, Mini Nutritional Assessment; MOS, Medical Outcomes Study; N, Number of patients; NCI, National Cancer Institute; NIA, National Institute on Aging; NSI, Nutrition Screening Initiative; PACE, Pre-operative assessment of cancer in the older; QOL, Quality of Life; SIC, Satariano’s Index of Co-morbidities.

The following aspects of each study were examined: date of publication; context of result; level of evidence the study presented (Level); number of participants (N); lower age cut-off of participants; type of cancer the participants had; stage of cancer; uniqueness of study; overall findings.

Level of evidence was assessed using the system proposed by Harbour and Miller [21], where evidence is graded in terms of ‘quantity, quality, consistency, applicability, generalizability and impact.’ This system has been adopted by the National Institute of Clinical Excellence [22]: ‘Grading evidence and recommendations for public health interventions’, considering factors including evidence of efficacy, cost effectiveness, research design, relevance to the UK population and consistency.

## Results

Nine full-text articles met our search criteria and all graded Level 3 for quality of evidence.

Despite the aim of this review to evaluate CGA in early operable breast cancer, we found a minimal number of studies being this specific, so all studies which contained any number of early breast cancer patients are discussed.

1. A pilot study by Extermann *et al.*[12], recruited patients from a single center aged ≥70 years with stage I or II breast cancer, after surgery. Baseline assessment was carried out and CGA completed on follow-up at three andsix months for 15 patients.

After CGA, cancer treatment was adjusted in four participants (36%); adjuvant endocrine therapy was selected in two patients and adjuvant chemotherapy in one patient. No information is available on the fourth patient.

In addition, CGA addressed problems indirectly impacting on treatment, in a further six patients (55%), for example, patient cognition, social support and contra-indicating medications. These problems were effectively resolved.

This study is unique in using the Functional Assessment of Cancer Treatment-Breast (FACT-B) instrument to measure QOL, validated by Extermann *et al.*[12]. Measures used in other similar studies (Table 1) are also incorporated, allowing comparisons to be made.

Due to the small number of patients (N = 15), findings from this study may not be comparable to all older primary breast cancer patients. Thus, more patients need to be recruited from several centers, to verify findings.

2. A pilot study by Hurria *et al.*[13], consisting of 40 patients from two institutions, developed and measured use of a cancer-specific geriatric assessment. Patients aged ≥65 years, with breast, lung, colorectal carcinoma or lymphoma, receiving chemotherapy and fluent in English, were recruited. The CGA was carried out after diagnosis with an aim to assess practicability of administration.

Average completion time was 27 minutes (range 8 to 45 minutes) and 78% of the patients were able to complete with no assistance. Approximately 90% of participants were happy with the questionnaire length and 83% agreed it was easy to understand.

This study used a cancer-specific geriatric assessment and has proven this is feasible. Of the 40 participants, 25% had breast cancer. The percentage of patients with primary operable cancer is unknown; findings specific to these patients cannot be determined.

The potential of applying this cancer-specific geriatric assessment tool could be evaluated in a multicenter study.

3. A prospective study by Pope *et al.*[14] recruited 460 patients from centers in the UK, Italy, the Netherlands, Belgium and Japan. Patients aged ≥70 years, undergoing surgery for cancer were included. The majority of the questionnaire was carried out prior to surgery with additional measures dependent on the outcome of surgery, completed afterwards.

Pope *et al.*[14] used an extended version of standard CGA in their study: Pre-operative Assessment of Cancer in the Older (PACE). In addition to the typical components of CGA, PACE includes supplementary information (Table 1) assessing overall functional performance.

As age of the patient increased, functional status decreased. There was less comorbidity among breast patients, when compared to those with gastro-intestinal tumors (GIT) and genito-urinary tumors (GUT). This could be due to the large number of patients with early breast cancer included in this study, compared with GIT and GUT, which consisted of patients with more evenly distributed cancer stages; patients in later stages may experience more severe symptoms. Alternatively, this could be explained by gender; greater comorbidity may exist in the male rather than female population; breast cancer patients are mainly female and GIT and GUT patients largely male.

This is an excellent international study using a large number of patients. All patients were receiving surgery, however only 47% for breast cancer, 87.3% being primary cases. Results appropriate to GIT and GIT cannot be differentiated from breast tumors in this study.

4. Albrand and Terret [15] conducted a study in a single unit for consecutive patients aged ≥70 years, presenting with primary breast cancer. The study employed CGA to detect medical risks influencing cancer management at follow up, for example, 17% of the cohort wwas at risk of cognitive deterioration and 30% of depression. These factors may impinge on the ability to make treatment decisions, or increase susceptibility to adverse treatment effects. These patients often present with good performance status so may be inadequately prescribed treatment based on this alone.

This study suggests CGA components related to function, mentality, nutrition and comorbidity assist in determining fitness for oncological treatments. Comorbidity was measured using the Cumulative Index Rating Scale-Geriatric (CIRS-G), which is only used by this study and the study by Extermann *et al.*[12] and, therefore, these studies cannot be directly compared to studies routinely using the Charlson listing [23].

Similar studies need to be conducted on a larger scale in multiple centers. Comparison of participants to matched patients not receiving CGA would be useful to determine if patient factors are acknowledged due to CGA or by increasing awareness of the patient’s own disease status.

5. A prospective, transversal study by Gironés *et al.*[16] was conducted in a single center to assess comorbidity in breast cancer survivors. Participants were aged ≥70 years and had primary operable breast cancer. The questionnaire was administered to 91 patients at follow-up.

The study showed these older breast cancer survivors were able to maintain function, but had high comorbidity; consequently, long-term follow-ups are recommended for cancer survivors. Gironés *et al.*[16] suggest multidimensional geriatric assessment (MGA) ,which considers the most relevant aspects of standard CGA only; thus it is shorter.

Similarly, it would be useful if patients in this study were matched to patients not receiving CGA.

6. A cross-sectional observational study by Molina-Garrido and Guillen-Ponce [17] was concerned with the feasibility of CGA application in early breast cancer patients. Between 1 January 2007 and 31 December 2007, 41 consecutive patients aged ≥65 years were recruited from a single center. All patients completed the Barber Questionnaire (BQ), the Vulnerable Elderly Survey (VES-13) and CGA, prior to receipt of chemotherapy.

This CGA showed correlation to the briefer measures of BQ and VES-13; patients who had a score indicative of frailty on CGA were more likely to score a high level of frailty on BQ and VES-13. Therefore, there is potential to develop a screening tool for administration of CGA. This study suggests CGA should be implemented when VES-13 score is <3 (maximum score 10, indicating best possible outcome).

Of 41 patients, 56.1% had no daily medications and no one had more than three daily medications, indicating a possible level of good health in this cohort; it is expected that older people will have greater comorbidity and thus more daily medications. Furthermore, 78% of participants were married, which is a larger proportion relative to others studies [13] and, hence, could be indicative of a high level of social support in this cohort, which is not always present in this age group. This sample may not be representative of the whole population with early breast cancer.

7. A retrospective study in a single center conducted by Barthélémy *et al.*[18] attempted to assess the impact that geriatric assessment, age and other prognostic factors had on treatment proposal of chemotherapy. All patients with early breast cancer ≥70 years discussed by the breast cancer tumor board at the University Hospital of Strasbourg, between July 2006 and July 2009, were considered. Patients were excluded if they presented with either metastatic or a recurrence of breast cancer, as well as those with a history of other cancer or previous chemotherapy. All patients between age 70 and 79 with at least one comorbidity, as well as all patients aged ≥79 years, had been referred for CGA after discussion by the breast cancer tumor board and thus there were a total of 192 patient records available for analysis.

Prognostic factors, for example, estrogen and progesterone receptor status, and tumor stage were recorded. The CGA included elements focusing on the domains of comorbidity, mood, medication, social support, environmental assessment, nutritional status and motor function.

In this sample, 118 out of the 192 patients had at least one or more risk factors which would ordinarily justify the use of adjuvant chemotherapy. However, only 70 of these patients (59%) were actually recommended adjuvant chemotherapy after discussion with the multidisciplinary team. The patients who did not receive chemotherapy despite showing good indications, received adjuvant endocrine therapy as an alternative.

Barthélémy *et al.*[18] concluded that age was the only independent factor associated with a lower rate of adjuvant chemotherapy recommendation in this sample of patients, especially after the age of 80 years. It is suggested that CGA is useful in identifying patients who may be at risk of adverse effects of chemotherapy, but not necessarily identifying those who may benefit from chemotherapy.

It is, however, worth noting that this study is largely regarding administration of chemotherapy, which is often not necessary for primary operable breast cancer cases, rather than assessing these patients at diagnosis aiming to formulate a management plan for primary therapy.

8. Lazarovici *et al.*[19] carried out a retrospective review of consecutive older patients refered to a single oncogeriatric unit from October 2006 to April 2008. A total of 65 patients were found and all had received CGA. The review aimed to establish the difference between those patients who had undergone CGA before treatment decision had been made (n = 35) and those after treatment of cancer had started (n = 30).

The CGA was conducted by a single geriatrician with oncological training. Where CGA was carried out before a treatment decision was made, this was done on the patient’s first visit to the geriatrician. The CGA assessed functional status, cognition, mood, nutritional status and comorbidity.

Recent weight loss of >10% was more frequent among the group of patients who had geriatric assessment before cancer treatment decision had been made (*P* = 0.031). These patients were subsequently later taking fewer medications (*P* = 0.036) and more likely to received adjusted cancer treatment (*P* = 0.051).

Lazarovici *et al.*[19] conclude that weight loss was the main feature leading to geriatric referral. Conducting CGA before treatment decision had been made resulted in a more personalized individual treatment plan for these patients.

A large proportion of patients in this study (60%) had metastatic disease. Therefore, there might be some selection bias in this study, as patients with primary operable breast cancer alone would have been less likely to be referred to the geriatrician under the criteria used in this study, and thus would not have undergone CGA.

9. A further multicenter study by Hurria *et al.*[20] aimed to identify risk factors for chemotherapy toxicity in older breast cancer patients, assessing many diagnostic and prognostic factors, including use of CGA. A total of 500 patients aged ≥65 years attending an oncology outpatient appointment at one of seven participating centers, between November 2006 and November 2009, were recruited to the study. All patients had a diagnosis of cancer and were scheduled to receive adjuvant chemotherapy.

Geriatric assessment was carried out before chemotherapy began. This study uses the same form of cancer-specific CGA as previously mentioned in the earlier study by Hurria *et al.*[13].

Regarding geriatric assessment, functional status, level of social activity, poor hearing and assistance required to take own medications, were important factors when considering chemotherapy toxicity.

This study was conducted on a large scale with the aim to identify any general factors relevant to all cancer types and stages with regards to toxicity from chemotherapy. The study did not look at whether there were any additional or different factors based on particular cancer types or stages.

## Discussion

Due to the heterogeneity of our sample papers, it is difficult to draw comparisons relating to our original aim of evaluating CGA use in early breast cancer.

The studies by Extermann *et al.*[12], Albrand and Terret [15], Gironés *et al.*[16] Molina- Garrido and Guillén-Ponce [17] and Barthélémy *et al.*[18] were solely concerned with primary operable breast cancer and, thus, have the greatest weight. However, these studies used a small (N <200) sample size in their investigations compared to the larger study by Pope *et al.*[14] (N = 460). In addition, these studies were conducted at a single-center only, whereas Pope *et al.*[14] conducted an international study, eliminating selection bias.

All studies used CGA to recognize comorbidities and significant factors present in their patients, which could potentially impact on treatment recommendation. The studies by Extermann *et al.*[12], Hurria *et al.*[13] and Gironés *et al.*[16] looked at CGA as a follow-up tool. The aim of this was to establish toxicity or problems arising from current treatment and determine necessary treatment changes. A further aim of the studies by Hurria *et al*. [13] and Molina-Garrido and Guillén-Ponce [17] was to assess feasibility of including older patients in clinical trials in general. The studies by Barthélémy *et al.*[18], Hurria *et al.*[13] and Hurria *et al.*[20] were primarily concerned with patients receiving chemotherapy and possibly toxicity arising from this.

All studies were Level 3 grade of evidence; conclusions made may not be strong enough to require immediate change to clinical practice [21,22].

Most of the studies used a similarly designed CGA, excluding the studies using cancer-specific CGA, by Hurria *et al.*[13,20]. The standard components of a non-cancer-specific CGA are not only used in cancer but in other areas of geriatric medicine also. More studies need to be conducted to determine whether a cancer-specific CGA is more reliable and accurate than a general CGA.

A number of studies [12,13,15] imply CGA can be used to determine treatment, especially regarding chemotherapy. Chemotherapy is not widely used in older people due to possible toxicity [24,25]. A CGA of the older cancer patient cannot only help establish fitness for surgery, but also whether adjuvant chemotherapy may be a viable option. The same reasoning applies to other cancer treatments.

It appears that of CGA may be difficult to complete due to impediments present in the older population in general [26,27]. This may be indicative of the type of treatment these patients should receive, or could simply be a hindrance on completion of the CGA by the patient, thus providing invalid data.

Due to the focused nature of this review, it appears that a number of important articles may have been excluded by our criteria, which are now discussed here.

Girre *et al.*[28] recruited 105 cancer patients aged ≥70 years for geriatric assessment. The majority of cancers were breast. Assessment of functional status, nutritional status, mood, physical function, medication and social and environmental support was made. Modification of treatment decision due to geriatric assessment was recorded in 39% of the patients. Factors associated with modification of treatment plan included body mass index and absence of depressive symptoms. Although no information on the exact changes to treatment plan is available, it is interesting that factors which may not be considered without application of CGA, may affect treatment decisions.

Also, an update of the study by Pope *et al.*[14] was written by Audisio *et al.*[29]. In addition to the previous report, they further analyzed 30-day morbidity and mortality and length of hospital stay in breast, GIT and GUT patients. In all groups, those experiencing complications were more likely to have a poor outcome on the assessment, specifically concerning comorbidity and physical functioning. Impairments of activities of daily living were important for predicting length of hospital stay. Pope *et al.*[14] and Audisio *et al.*[29] underline the impact of geriatric assessment but also suggest that a cancer-specific geriatric assessment for different subtypes of cancer should evolve.

## Conclusions

From the literature, there is not yet enough evidence to recommend CGA in early breast cancer patients. Currently, literature suggests that CGA may be useful in regard to treatment decision making in older cancer patients. This is consistent with the clinical and pilot research experience of the authors [30].

This literature review is hampered by lack of evidence currently available concerning use of CGA in early breast cancer patients. Analysis of some studies was inhibited by the extent of information available, resulting in difficultly in drawing comparisons between studies. Evidence so far suggests that CGA is an important factor in determining treatment and management of early breast cancer by identifying confounding health and personal issues of the patient and their suitability for different treatments, where this is possible. Case–control and cohort studies need to be completed to compare outcomes of patients who receive CGA to those who do not.

## Abbreviations

BQ = Barber Questionnaire; CGA = Comprehensive Geriatric Assessment; CIRS-G = Cumulative Index Rating Scale-Geriatric; FACT-B = Functional Assessment of Cancer Treatment-Breast; GIT = Gastro-intestinal tumours; GUT = Genito-urinary tumours; MGA = Multidimensional geriatric assessment; PACE = Pre-operative Assessment of Cancer in the Elderly; QOL = Quality of life; VES-13 = Vulnerable Elderly Survey.

## Competing interests

The authors declare that they have no competing interests.

## Authors’ contributions

RMP carried out the literature research, data acquisition, data analysis and prepared the manuscript. KLC is the guarantor of the integrity of the study and defined the intellectual content. KLC, DALM and KC created the study concept and design. All authors edited the manuscript and read and approved the final manuscript.
